# Development of microsatellite markers for *Primula oreodoxa* (Primulaceae), a distylous‐homostylous species

**DOI:** 10.1002/aps3.1150

**Published:** 2018-05-23

**Authors:** Shuai Yuan, Gui Zeng, Dianxiang Zhang

**Affiliations:** ^1^ Key Laboratory of Plant Resources Conservation and Sustainable Utilization South China Botanical Garden Chinese Academy of Sciences Guangzhou 510650 People’s Republic of China; ^2^ University of the Chinese Academy of Sciences Beijing 10049 People’s Republic of China

**Keywords:** distylous‐homostylous, mating system, microsatellites, *Primula oreodoxa*, Primulaceae, RAD sequencing

## Abstract

**Premise of the Study:**

Microsatellite markers were developed for a distylous‐homostylous species, *Primula oreodoxa* (Primulaceae), to investigate the mating patterns and gene flow in the species.

**Methods and Results:**

Using RAD sequencing, 42,777 contigs were generated. A total of 1566 putative simple sequence repeat (SSR) loci were identified by MISA, and 1433 primer sets were designed. After initial screening of 107 SSR loci, 24 loci displayed polymorphism. The number of alleles per locus detected ranged from one to 11 in the sampled populations of *P. oreodoxa*. Levels of observed and expected heterozygosity for each locus ranged from 0 to 0.917 and 0 to 0.816, respectively. Fifteen and 13 of these loci could be successfully amplified in the two congeneric species *P. obconica* and *P. heucherifolia*, respectively.

**Conclusions:**

The SSR markers developed here will be valuable for genetic analysis and elucidation of mating patterns in *P. oreodoxa*.

Evolutionary breakdown from distyly to homostyly represents a reproductive transition from outcrossing to selfing, resulting in important ecological, genetic, and evolutionary consequences (Barrett and Shore, 2008). Detection of the outcrossing and selfing rates of populations with different floral types will improve understanding of how mating systems change (Runquist et al., 2017). The widespread availability of co‐dominant genetic markers (simple sequence repeats [SSRs]) has enabled refined estimation of the mating system of plants using paternity assignment (Karron et al., 2012). *Primula oreodoxa* Franch. (Primulaceae) is a herbaceous perennial endemic to the mountains of western Sichuan, China (28°–31°N, 102°–104°E). There are three types of populations in *P. oreodoxa*: distylous (with long‐ and short‐styled morphs), homostylous, and mixed (with long‐ and short‐styled, and homostylous morphs) populations. The complex pattern of floral variation provides a model system to investigate the genetic mechanism associated with mating system transitions (Yuan et al., 2017a). By using microsatellites, we can quantify the contribution of female and male function for each floral morph. Although a certain number of genomic SSR markers have been developed for several *Primula* L. species, such as *P. vulgaris* Huds. (Van et al., 2006), *P. veris* L. (Bickler et al., 2013), *P. poissonii* Franch. and *P. wilsonii* Dunn (Zhang et al., 2013), and *P. ovalifolia* Franch. (Yuan et al., 2017b), most of them show limited transferability to *P. oreodoxa* because *P. oreodoxa* is phylogenetically distant from these species. In our phylogeographic study of *P. oreodoxa* (Yuan et al., 2017a), only four markers previously developed from *P. sieboldii* E. Morren (Ueno et al., 2009) demonstrated sufficient polymorphism to provide precise estimation of mating systems. Thus, additional SSR markers specific to *P. oreodoxa* are needed to provide useful tools for evolutionary and ecological studies on the species. In this study, we describe the method of isolation and development of SSR markers for *P. oreodoxa* using RAD sequencing. Some of the markers were selected to investigate their polymorphism in *P. oreodoxa* and transferability to two congeneric species, *P. obconica* Hance and *P. heucherifolia* Franch., which are phylogenetically relatively close to *P. oreodoxa*.

## METHODS AND RESULTS

Total DNA of *P. oreodoxa* was extracted from fresh leaves of one individual sampled from population ORE_JCC (Appendix 1). Following Baird et al. (2008), we constructed a DNA library using a RAD sequencing method. By randomly shearing from samples, fragments with an average size of 500 bp were obtained (Bioruptor Branson Sonicator 450; Branson Ultrasonics, Danbury, Connecticut, USA). The harvested fragments were run on a 1% agarose gel (Sigma‐Aldrich, St. Louis, Missouri, USA), and only those with lengths ranging from 300 to 500 bp were selected for further analysis using the MinElute Gel Extraction Kit (QIAGEN, Hilden, Germany). Subsequently, library sequencing was performed using the Illumina HiSeq X Ten platform (Illumina, San Diego, California, USA). Reads were filtered by trimming adapter sequences and removing reads containing ambiguous or low‐quality bases (Phred score < Q10). Raw reads were obtained and deposited in the National Center for Biotechnology Information Short Read Archive (BioProject ID PRJNA417906; accession no. SRA6281248). The quality‐filtered RAD reads were assembled using the SOAP2 program with 97 *k*‐mers (Li et al., 2009), and 42,777 contigs were built. The SSR markers were then identified from the contig set (≥100 bp) using the MIcroSAtellite identification tool (MISA) based on the Perl language (Thiel et al., 2003). We searched for SSRs with motifs ranging from mono‐ to hexanucleotides in size. The SSR length criteria were defined with 10 repeat units for mononucleotide, at least six iterations for dinucleotide repeats, and at least five for tri‐, tetra‐, penta‐, and hexanucelotide repeats. Then, 1433 primer pairs were designed from 1566 putative loci using Primer3 web version 0.4.0 (Untergasser et al., 2012; http://bioinfo.ut.ee/primer3-0.4.0/primer3/).

During the preliminary test with four individuals of *P. oreodoxa*, 107 dinucleotide motifs containing at least eight contiguous repeat units were screened, and 82 primers produced clear bands with suitable fragment lengths by agarose gel electrophoresis (<500 bp). Eight individuals from four different populations were then employed for further detection using these 82 loci. Genomic DNA of populations was extracted using a modified cetyltrimethylammonium bromide (CTAB) method (Doyle, 1991). A forward primer with an M13(–21) tail at its 5′ end, a reverse primer, and the universal fluorescent‐labeled M13(–21) primer (FAM, ROX, HEX, or TAMRA; Invitrogen, Guangzhou, China) were included in the PCR reactions (Schuelke, 2000). The amplified 10‐μL mixture included 5 μL of Master Mix (Generay Biotech, Guangzhou, China), 0.4 mM of each primer, 3.2 μL of deionized water, and 30–50 ng of genomic DNA. A touchdown procedure was performed for all loci with initial denaturation for 4 min at 94°C; followed by 10 cycles of 94°C for 35 s, 35 s at 60°C with an increment of −1°C per cycle, and 45 s at 72°C; followed by 28 cycles of 94°C for 35 s, 35 s at 50°C, and 45 s at 72°C; and an extra extension of 10 min at 72°C. PCR products of every four loci with different dyes (see Table 1) were mixed together and scanned by an ABI PRISM 3100 Genetic Analyzer (Invitrogen) using a GeneScan 500 LIZ internal size standard (Thermo Fisher Scientific, Waltham, Massachusetts, USA). Allele binning and calling were done using GeneMarker version 2.4.0 (SoftGenetics, State College, Pennsylvania, USA). After discarding the loci that were monomorphic or did not amplify cleanly or consistently, we obtained 24 polymorphic loci with at least two alleles per locus (Table 1). All of these SSR sequences have been deposited in GenBank (Table 1).

**Table 1:** Characteristics of 24 microsatellite loci identified in *Primula oreodoxa*.^a^

Locus	Primer sequences (5′–3′)	Repeat motif	Allele size range (bp)	Fluorescent dye	GenBank accession no.
c8899	F: GCGAGGGGTTGTGTTGTTTA	(TG)_11_	194–204	FAM	MG324209
	R: TGCCTTGTGTCAGTTTTGGT				
c8979	F: AACATCAGAGTGACGGTTTGG	(TC)_13_	243–249	HEX	MG324210
	R: GGGTTTGAGAGAGAAGCAATTT				
c12873	F: GCATGAAAATGCGAAGACAA	(AG)_8_	185–219	TAMRA	MG324211
	R: GGGTGGGGGAGTAAAGAAGA				
c14497	F: TATTGATCTCAGCAAGGGGG	(AG)_8_	154–158	ROX	MG324212
	R: TCAACCAGAGTTGCTCTTTACTTTT				
c15305	F: CTGAATCCCCTAACTTTGCG	(AG)_11_	214–236	FAM	MG324213
	R: TTTCACCACTGACGATCGAG				
c27661	F: TCCATCGCTATTTCTGCTTG	(GA)_13_	205–217	HEX	MG324214
	R: CCCTAAATAAATCGACGGTGA				
c28817	F: TAAACAAGTTCGCGCTTCCT	(TC)_8_	172–180	TAMRA	MG324215
	R: AAACCCTAAAATTATTCAGATCGAGA				
c30367	F: TAGGGTTTTTCCTCCCTTGC	(TC)_16_	168–184	ROX	MG324216
	R: CAATTTTACCATTCGTGCCC				
c34171	F: GTGGGTCGATATGAGCGAGT	(TC)_17_	132–184	FAM	MG324217
	R: TGTCTTTCCCCACCGACTTA				
c39209	F: AAATCCCTCCGTAACAAACG	(TC)_11_	258–300	HEX	MG324218
	R: TTTGGTCGTCCACGTAATCA				
c40223	F: GGGGAAAATTAAGGTACGGC	(GT)_8_	176–192	TAMRA	MG324219
	R: TGCAGCCATATTTTGGAGAA				
c42193	F: TTTTCCCATAACGGTGCTTC	(CT)_10_	206–284	ROX	MG324220
	R: CGAACTGCCATAGTTAGGCG				
c42439	F: AAACACTACACTTCACTCCTCTCC	(CT)_12_	160–204	FAM	MG324221
	R: TGGGATGGAAACATAGCCTC				
c45817	F: TTGCACCTTTCTTCATCGTG	(CA)_10_	175–193	HEX	MG324222
	R: CAGCAGCACCTGCAACTAGA				
c51771	F: GGTAGTTTCGGGTCAAGCAA	(CT)_11_	204–228	TAMRA	MG324223
	R: GCGATGGGGAGTAAAGTTGA				
c52063	F: GTGTGGGTGAAACCGAACTC	(GA)_8_	148–158	ROX	MG324224
	R: ACCACATCGATCTCCTCCG				
c54489	F: TGTTGAGGAGCCCCATGTAT	(GA)_8_	190–204	FAM	MG324225
	R: TAAAGCGACAATTGGCACAG				
c54691	F: GGAAAAACGGCTTCATTCAA	(TC)_9_	204–226	HEX	MG324226
	R: GAGCGTCTCCGTTGCTAGG				
c61111	F: CTAACGGGGAGGCCTAAAAG	(CT)_14_	251–273	TAMRA	MG324227
	R: GTTGTTGGGCAAAAGGAAAA				
c62717	F: ACATCGTCCGTTTCTCATCA	(AG)_11_	267–309	ROX	MG324228
	R: TGACAGGGACCATTTCATCA				
c62889	F: TCACGATCCTAATTCACCCC	(CT)_9_	272–290	FAM	MG324229
	R: ACACGTCATCAAGTGGGTCA				
c66597	F: ACCAATATTTGTTGCCCAGC	(TC)_8_	220–230	HEX	MG324230
	R: CCACTTTTGGGGGTTTCTTC				
c75429	F: AGAGACCCACCTCCTGGTCT	(AG)_17_	232–280	TAMRA	MG324231
	R: TAAATGGGGGATACAGCCAA				
c80317	F: GGAAAAAGGGAGGCTTATCAA	(TC)_9_	184–200	ROX	MG324232
	R: GTCGGATAGTCGTCGGTGTT				

a A touchdown program was performed for all loci with annealing temperature from 60°C to 50°C.

### Polymorphism and transferability assessment

The 24 selected loci were further assessed in three populations of *P. oreodoxa*, one of *P. obconica*, and one of *P. heucherifolia* (Appendix 1). GenAlEx 6.5 (Peakall and Smouse, 2012) was used to calculate the number of observed alleles per locus, expected heterozygosity, observed heterozygosity, and linkage disequilibrium among loci per population. By employing FSTAT 2.9.3, we tested the deviation from Hardy–Weinberg equilibrium for each locus (Goudet, 2001). We also tested the presence of null alleles using MICRO‐CHECKER software (van Oosterhout et al., 2004).

Genetic analysis revealed no significant linkage disequilibrium among loci after Bonferroni correction at a confidence level of α = 0.05. However, significant deviations from Hardy–Weinberg equilibrium were observed for half of these loci (Table 2). Results from MICRO‐CHECKER indicated the possible presence of null alleles in some of the loci. However, an excess of homozygotes in these loci may result from self‐pollination because *P. oreodoxa* is completely self‐compatible and has a pollinator‐limited environment (Yuan et al., 2017a). The average number of alleles detected was 4.8 (range 2–8), 4.5 (range 1–10), and 5.3 (range 1–11), respectively, in the three populations of *P. oreodoxa*. Levels of observed and expected heterozygosity for each locus ranged from 0 to 0.917 and 0 to 0.834, respectively. Among the 24 SSR markers, 15 loci were successfully amplified, with at least two alleles per locus in *P. obconica* (Table 2). Similarly, 13 loci showed polymorphism in *P. heucherifolia* (Table 2).

**Table 2:** Results of initial primer screening in populations of three *Primula* species.^c^

Locus	*P. oreodoxa*												*P. obconica*				*P. heucherifolia*			
	ORE_JCC				ORE_DWS				ORE_QLP				OBC_MTG				HEU_LWP			
	*N*	*A*	*H*_o_	*H*_e_	*N*	*A*	*H*_o_	*H*_e_	*N*	*A*	*H*_o_	*H*_e_	*N*	*A*	*H*_o_	*H*_e_	*N*	*A*	*H*_o_	*H*_e_
c8899	23	4	0.478	0.722	24	6	0.458	0.680^a^, ^b^	24	4	0.375	0.650		—	—	—	24	3	0.583	0.588
c8979	24	3	0.000	0.448^a^, ^b^	24	3	0.542	0.602	24	4	0.250	0.353	20	1	0.000	0.000	14	3	0.000	0.255^a^, ^b^
c12873	23	6	0.348	0.733^a^, ^b^	24	3	0.750	0.624	24	7	0.375	0.717^a^, ^b^	12	7	0.286	0.824^a^, ^b^	20	7	0.250	0.830^a^, ^b^
c14497	24	2	0.042	0.041	24	1	0.000	0.000	24	1	0.000	0.000	24	2	0.000	0.080^a^, ^b^	24	1	0.000	0.000
c15305	24	5	0.333	0.678	24	5	0.667	0.663	24	7	0.375	0.811^a^		—	—	—		—	—	—
c27661	24	4	0.208	0.650^a^, ^b^	23	4	0.609	0.627^a^	23	6	0.565	0.749^a^, ^b^		—	—	—		—	—	—
c28817	24	3	0.167	0.517^a^, ^b^	24	4	0.583	0.654	24	2	0.125	0.457^a^, ^b^	10	3	0.100	0.265^a^	11	2	0.364	0.397
c30367	22	6	0.591	0.753	24	2	0.417	0.330	24	5	0.667	0.712		—	—	—		—	—	—
c34171	24	3	0.625	0.452	24	4	0.458	0.522^a^	24	5	0.625	0.497^a^	23	2	0.000	0.083^a^	24	4	0.208	0.194
c39209	24	6	0.208	0.533^a^, ^b^	24	6	0.542	0.611^a^	24	9	0.542	0.834^a^, ^b^	18	9	0.333	0.758^a^, ^b^				
c40223	23	5	0.417	0.641^a^, ^b^	24	5	0.250	0.543^a^, ^b^	24	4	0.417	0.622	11	4	0.545	0.640	17	6	0.529	0.697^a^
c42193	24	8	0.739	0.763^a^	23	10	0.522	0.816^a^, ^b^	22	8	0.455	0.782^a^, ^b^		—	—	—		—	—	—
c42439	24	6	0.292	0.650^a^, ^b^	24	6	0.333	0.738^a^, ^b^	24	6	0.458	0.685^a^, ^b^	22	3	0.273	0.459^a^	24	7	0.375	0.569^a^, ^b^
c45817	24	7	0.500	0.757^a^, ^b^	24	4	0.250	0.293^a^	24	2	0.125	0.117	21	5	0.286	0.495^a^, ^b^		—	—	—
c51771	24	7	0.375	0.554^a^, ^b^	23	6	0.391	0.682^a^, ^b^	24	8	0.500	0.742	10	4	0.600	0.615		—	—	—
c52063	24	3	0.417	0.455	24	2	0.458	0.478	24	3	0.458	0.588	19	2	0.105	0.100		—	—	—
c54489	24	4	0.333	0.393	24	6	0.792	0.677	24	4	0.500	0.607	13	4	0.231	0.521	22	3	0.188	0.525^a^
c54691	24	5	0.375	0.659^a^, ^b^	24	5	0.833	0.701	24	3	0.458	0.609	11	3	0.273	0.244	16	8	0.364	0.744^a^, ^b^
c61111	24	6	0.333	0.609^a^, ^b^	24	4	0.333	0.568	23	8	0.565	0.800		—	—	—		—	—	—
c62717	24	7	0.375	0.528	23	5	0.435	0.612	24	7	0.333	0.786^a^, ^b^		—	—	—		—	—	—
c62889	24	5	0.583	0.626^a^	24	4	0.583	0.565	24	4	0.167	0.355^a^, ^b^	8	5	0.375	0.719^a^, ^b^	13	5	0.308	0.393
c66597	24	3	0.792	0.546	24	2	0.917	0.500^a^	24	4	0.333	0.294	17	3	0.176	0.403	22	5	0.227	0.448^a^, ^b^
c75429	24	4	0.250	0.440	24	6	0.667	0.774^a^	24	11	0.292	0.808^a^, ^b^		—	—	—	23	5	0.435	0.426
c80317	24	4	0.292	0.568^a^, ^b^	24	5	0.458	0.668^a^, ^b^	24	6	0.417	0.502		—	—	—		—	—	—
Mean		4.8	0.378	0.572		4.5	0.510	0.580		5.3	0.391	0.586		3.8	0.239	0.414		5.3	0.302	0.471

Note:

— = unsuccessful amplification in this population; *A* = number of alleles; *H*_e_ = expected heterozygosity; *H*_o_ = observed heterozygosity; *N* = number of successfully amplified individuals.

a Significant deviation from Hardy–Weinberg equilibrium (*P* < 0.05).

b Significant possibility of the presence of null alleles.

c Locality and voucher information are provided in Appendix 1.

## CONCLUSIONS

In this study, we developed 24 SSR markers for *P. oreodoxa* based on RAD sequencing. These markers showed high polymorphism in three populations of *P. oreodoxa*. In the two congeneric species tested, more than half of the markers could be successfully amplified. These SSR markers will be useful tools for the investigation of mating pattern of each floral morph in both of the studied populations, as well as for broader evolutionary studies.

## APPENDIX 1 Locality and voucher information for populations of *Primula oreodoxa*,* P. obconica*, and *P. heucherifolia* used in this study. Voucher specimens are deposited at the herbarium of the South China Botanical Garden (CAS), Guangzhou, Guangdong, China.

**Table:**

Species	Population code	Floral polymorphism	Voucher no.	Location	Geographic coordinates	Altitude (m)	*n*
*Primula oreodoxa* Franch.	ORE_JCC	Distyly	YS231	E’mei, China	29°27′42″N, 103°13′07″E	1668	24

ORE_DWS	Distyly	YS509	Leshan, China	29°22′15″N, 103°01′12″E	1887	24

ORE_QLP	Distyly mixed with homostyly	YS417	Hongya, China	29°29′59″N, 103°14′40″E	1820	24
*Primula obconica* Hance	OBC_MTG	Distyly	YS251	E’mei, China	29°34′52″N, 103°22′04″E	1321	24
*Primula heucherifolia* Franch.	HEU_LWP	Distyly	YS333	E’mei, China	29°32′49″N, 103°20′24″E	2448	24
